# Effects of the Activin A–Follistatin System on Myocardial Cell Apoptosis through the Endoplasmic Reticulum Stress Pathway in Heart Failure

**DOI:** 10.3390/ijms18020374

**Published:** 2017-02-10

**Authors:** Miao Liu, Cuiying Mao, Jiayu Li, Fanglei Han, Ping Yang

**Affiliations:** 1 Department of Cardiology, China-Japan Union Hospital of Jilin University, Changchun 130031, China; anne20081122@126.com (M.L.); megeren@163.com (C.M.); yuanyuanlijiayu@163.com (J.L.); 2 Department of Anesthesiology, China-Japan Union Hospital of Jilin University, Changchun 130031, China; cian1984@163.com

**Keywords:** activin A, apoptosis, endoplasmic reticulum stress, follistatin, heart failure

## Abstract

Background: A previous study suggested that activin A inhibited myocardial cell apoptosis. This study thus aimed to explore the effects of the activin A–follistatin system on myocardial cell apoptosis in heart failure (HF) rats in order to determine whether or not the mechanism operates through the endoplasmic reticulum stress (ERS) pathway. Methods: Myocardial infarction (MI) by vascular deprivation was used to induce HF. The enzyme-linked immunosorbent assay was used to detect activin A, follistatin and brain natriuretic peptide (BNP) contents in serum. Immunohistochemical staining for activin A, follistatin, CCAAT-enhancer-binding protein (C/EBP) homologous protein (CHOP) and caspase-3 was performed on the myocardial tissue. The activin A-stimulated apoptosis of H9c2 cells was tested by flow cytometry. Western blot was used to detect the expression levels of activin A, follistatin and ERS-related proteins. Results: It was found that the high expression of activin A could cause activin A–follistatin system imbalance, inducing myocardial cell apoptosis via ERS in vivo. When HF developed to a certain stage, the expression of follistatin was upregulated to antagonize the expression of activin A. Activin A inhibited cardiomyocyte apoptosis with a low concentration and promoted apoptosis with a high concentration in vitro, also via ERS. Conclusion: Activin A–follistatin system participated in ERS-mediated myocardial cell apoptosis in HF.

## 1. Introduction

Activin is a subgroup of the transforming growth factor (TGF)-β superfamily. It consists of two disulfide-linked activin β subunits (βA–βD) [1]. Activin A is a homodimeric glycoprotein consisting of two βA subunits, which has a number of important functions in embryonic development, cell proliferation, differentiation, reproductive biology, fibrosis, apoptosis, metabolism, homeostasis, immune response, wound repair, inflammation, and so on [1,2,3,4,5,6,7,8,9,10,11,12,13,14,15]. Follistatin is widely expressed in various tissues as an extracellular antagonist preventing activin from binding to activin receptor (ActR) [16,17]. Activin A and follistatin are usually expressed in tissues and organs at the same time. The imbalance of activin A–follistatin system participates in remodeling and repairing of tissues. For instance, a few studies showed that the over-expressed activin A in the skin of transgenic mice led to dermal fibrosis and excessive epidermal incrassation, whereas the over-expressed follistatin distinctly reduced the formation of the scar areas [5,18].

Activin A can cause apoptosis. For example, activin A can inhibit DNA synthesis induced by mitogen, so as to inhibit the growth of liver cells and proliferation of liver [19] and induce liver cell apoptosis in vitro and in vivo [20,21]. Cell apoptosis was activin A dose dependent within a certain range. A recent study, however, has suggested that activin A could inhibit acute ischemia- and hypoxia-induced myocardial cell apoptosis [22], proving that activin A could induce or inhibit apoptosis. At the same time, whether activin A inhibits myocardial cell apoptosis after chronic damage such as heart failure (HF) after myocardial infarction (MI) needs to be confirmed. This study has therefore established an HF model after MI to observe the influence of activin A on chronic injured myocardial cells.

Endoplasmic reticulum stress (ERS) has a protective effect on myocardial cells when activated by stress as an initial response. However, upon excessive activation, it results in myocardial cell apoptosis initiated by the transcriptional activation of C/EBP homologous protein (CHOP) or c-JUN NH2-terminal kinase (JNK)- or caspase-12-dependent pathways [23,24]. ERS and activin A have the same influence at this time point. H9c2 cell is a subclone of the original clonal cell line derived from embryonic BD1X rat heart tissue by Kimes and Brandt, and exhibits many properties of skeletal muscle [25]. Myoblastic cells in this line will fuse to form multinucleated myotubes and respond to acetylcholine stimulation. H9c2 cells can split but not beat, and are usually used as an experimental model in vitro because they are confirmed to be a perfect alternative of primary neonatal cardiomyocytes for signal transduction studies [26]. Assuming that activin A participates in myocardial cell apoptosis through ERS, the expression of activin A–follistatin system and ERS-related molecules (glucose-regulated protein-78 (GRP-78), CHOP, and caspase-12) was tested in this study in rats with HF at different time points after MI. Angiotensin II (Ang II) has important autocrine and paracrine functions in a variety of organs. It plays an especially important role in HF [27,28,29]. A study reported that Ang II-activated ERS increased and could induce cell apoptosis [30]. Therefore, H9c2 cells were cultured in vitro, and Ang II was used to induce apoptosis in this study to investigate the relationship between the activin A–follistatin system and ERS.

## 2. Results

### 2.1. General Observations

**Figure 1:**
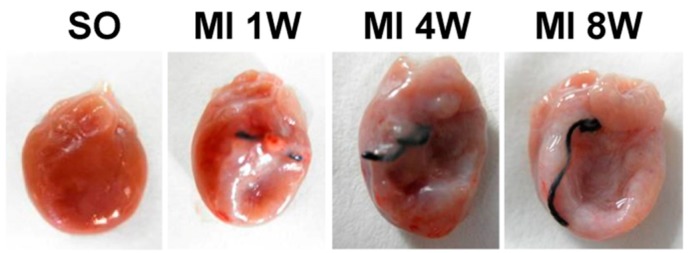
General observations.

The sizes of hearts very obviously increased in the four-week and eight-week MI groups compared to the sham-operated (SO) groups, and the longer the duration of MI, the more significant the change (Table 1). The ligated infarction areas appeared to be thinner compared to the noninfarction areas. A part of the infarction areas were nearly transparent, and the color of the junction progressively turned pale red (Figure 1).

### 2.2. Morphological and Hemodynamic Characteristics of Rats

The morphological and hemodynamic characteristics of rats were tested using the BCL-400E biological function experiment system (Table 2). The ratio of heart weight (HW) to body weight (BW) and the ratio of left ventricular heart weight (LVW) to BW were calculated to present the whole heart hypertrophy and left ventricular hypertrophy.

### 2.3. Expression Levels of Activin A–Follistatin System and Brain Natriuretic Peptide (BNP), Angiotensin II (Ang II) in the Serum of Rats

**Figure 2:**
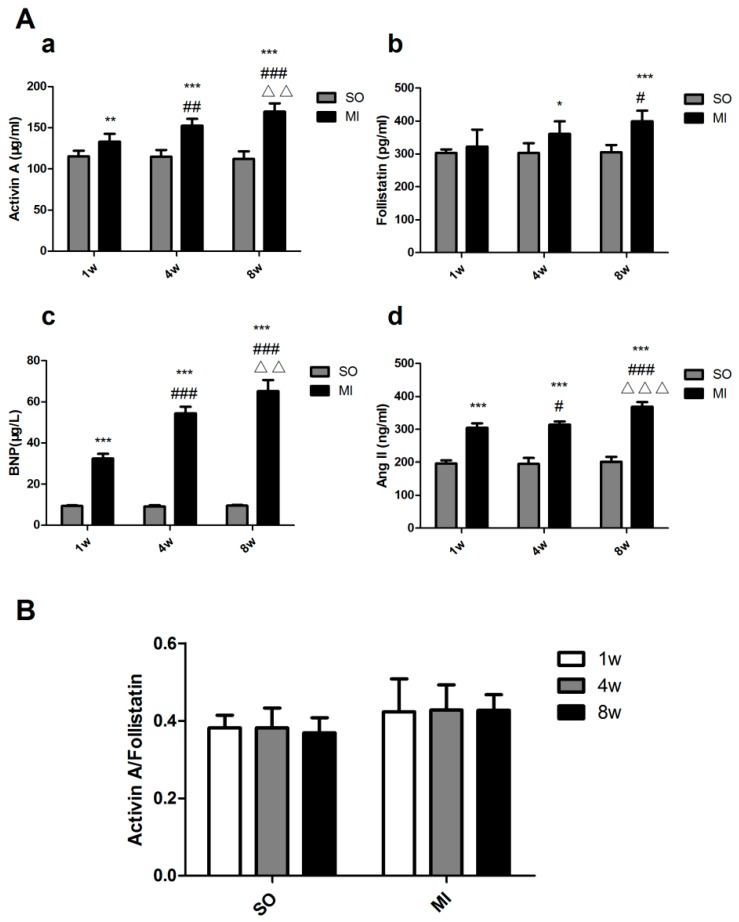
Expression levels of activin A–follistatin system, brain natriuretic peptide (BNP) and angiotensin II (Ang II) in the serum of rats. (**A**) ELISA method was used to determine the expression of activin A–follistatin system, BNP and Ang II. The data are presented as mean ± standard deviation. * *p* < 0.05, ** *p* < 0.01, *** *p* < 0.001, compared with the SO group. ^#^ *p* < 0.05, ^##^ *p* < 0.01, ^###^ *p* < 0.001, compared with the one-week MI group. ^ΔΔ^ *p* < 0.01, ^ΔΔΔ^ *p* < 0.001, compared with the four-week MI group; (**B**) Activin A/Follistatin represents the ratio of activin A and follistatin.

The one-week MI group displayed high expression levels of serum activin A, BNP and Ang II compared with the control group, with a significant difference (*p* < 0.01, *p* < 0.001, *p* < 0.001). The longer the duration of MI, the greater the expression of activin A, BNP and Ang II. The expression of follistatin started four weeks after MI (*p* < 0.05) and increased obviously in eight weeks after MI (*p* < 0.001) (Figure 2A). The ratio of activin A to follistatin in the serum of the same rats was also calculated, and the ratio was found to be nearly unchanged in the one-week MI group, the four-week MI group, and the eight-week MI group (*p* > 0.05) (Figure 2B).

### 2.4. Detection of the Expression of Activin A, Follistatin, Caspase-3, and C/EBP Homologous Protein (CHOP) by Immunohistochemical Staining

**Figure 3:**
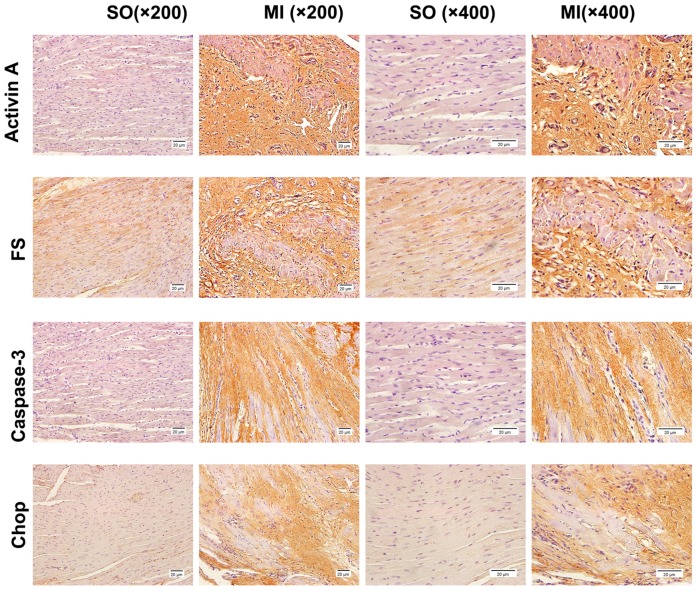
The expression of activin A, follistatin, caspase-3, and C/EBP homologous protein (CHOP) in rats detected by immunohistochemical staining. The border zones were used to take photos in the eight-week MI group. The expression of activin A, follistatin, caspase-3, and CHOP was detected using the anti-activin A antibody, anti-follistatin antibody, anti-caspase-3 antibody, and anti-CHOP antibody; IgG from a normal rabbit served as a negative control (200× magnification and 400× magnification).

Activin A, follistatin, and CHOP were expressed at low levels in the myocardial tissue of the control group; no caspase-3 was expressed. The expression of activin A, follistatin, caspase-3, and CHOP increased obviously in infarction and noninfarction areas of the myocardial tissue in rats with eight-week MI (Figure 3).

### 2.5. Detection of the Expression of Activin A, Follistatin, BNP, Caspase-3 and Endoplasmic Reticulum Stress (ERS)-Related Molecules in the Left Ventricular Noninfarction Area by Western Blot

**Figure 4:**
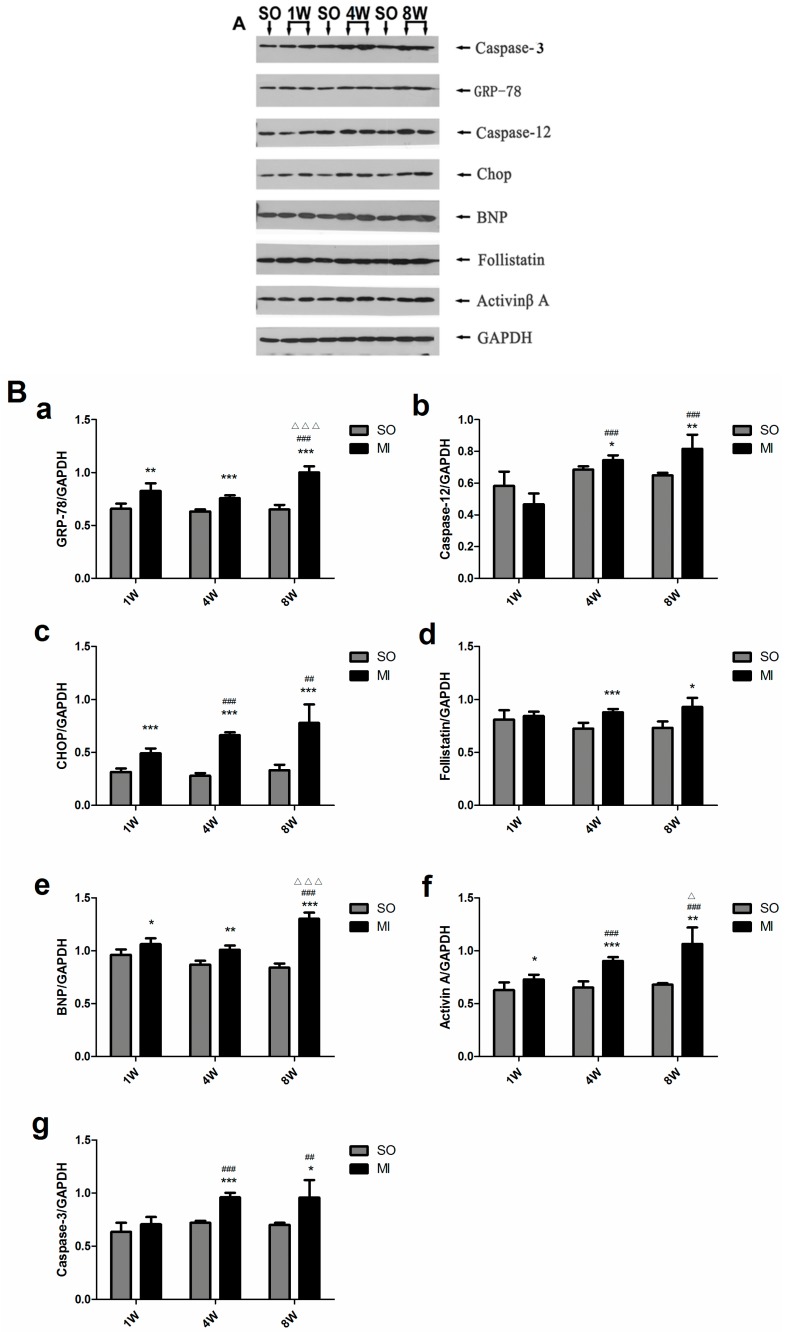

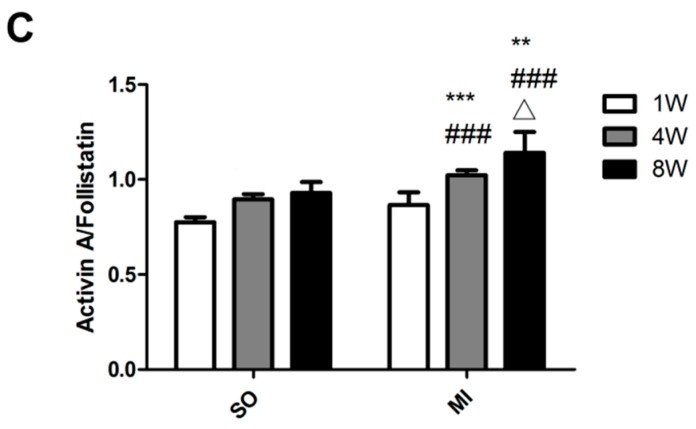
Expression levels of activin A, follistatin, BNP, caspase-3 and ERS-related molecules in the left ventricular noninfarction area. (**A**) The expression of activin A, follistatin, BNP, caspase-3, glucose-regulated protein-78 (GRP-78), caspase-12, and CHOP was detected by Western blot assay; (**B**) The graph shows the result of densitometry quantification of proteins relative to glyceraldehyde-3-phosphate dehydrogenase (GAPDH) as internal control. (**a**) GRP-78, (**b**) Caspase-12, (**c**) CHOP, (**d**) Follistatin, (**e**) BNP, (**f**) activin A, (**g**) Caspase-3; (**C**) Activin A/Follistatin represents the ratio of activin A and follistatin. The data are presented as mean ± standard deviation. * *p* < 0.05, ** *p* < 0.01, *** *p* < 0.001, compared with the SO group. ^##^ *p* < 0.01, ^###^ *p* < 0.001, compared with the one-week MI group. ^Δ^ *p* < 0.05, ^ΔΔΔ^ *p* < 0.001, compared with the four-week MI group.

GRP-78, CHOP, BNP, and activin A began to be highly expressed one week after MI (*p* < 0.01, *p* < 0.001, *p* < 0.05, and *p* < 0.05) compared with the control group. Caspase-3, caspase-12 and follistatin increased significantly in four weeks after MI (*p* < 0.001, *p* < 0.001 and *p* < 0.05). However, the expression levels of follistatin in the MI groups were nearly unchanged as the duration of MI extended (Figure 4A,B). The ratios of all MI groups increased compared with the SO groups, and the increases were more apparent as MI prolonged (Figure 4C).

### 2.6. Effect of Different Concentrations of Activin A on H9c2 Cell Apoptosis

**Figure 5:**
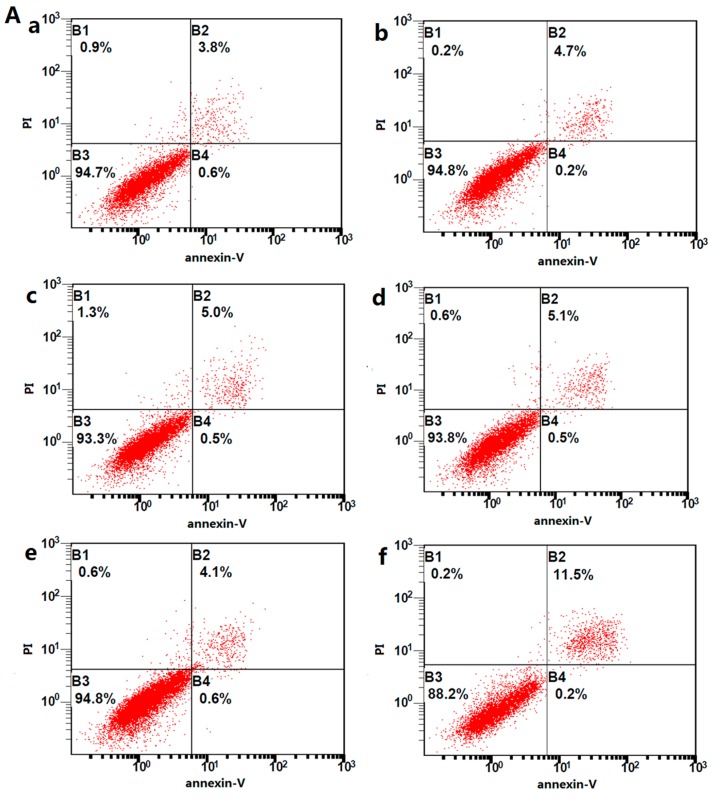

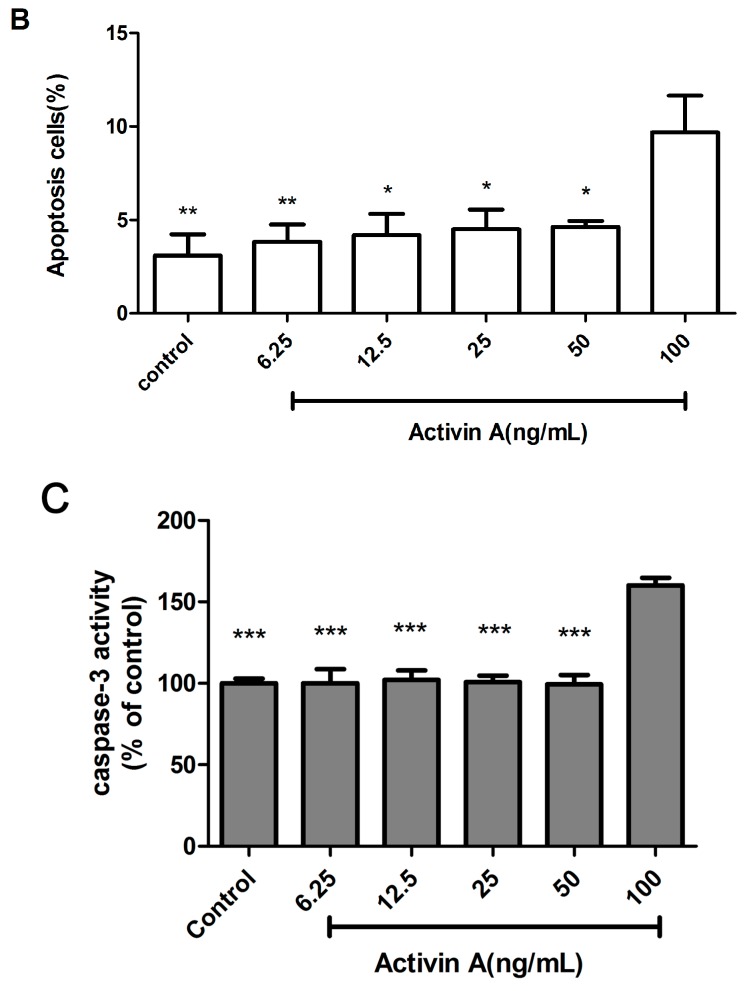
Apoptosis in activin A-stimulated H9c2 cells. (**A**) H9c2 cells were stained with propidium iodide (PI) and Annexin V–fluorescein isothiocyanate (FITC) and sorted by fluorescence-activated flow cytometry. (**a**) control; (**b**) activin A, 6.25 ng/mL; (**c**) activin A, 12.5 ng/mL; (**d**) activin A, 25 ng/mL; (**e**) activin A, 50 ng/mL; and (**f**) activin A, 100 ng/mL; (**B**) Graph presented the percentage of H9c2 cell apoptosis. The data are presented as mean ± standard deviation. * *p* < 0.05, ** *p* < 0.01 versus the 100 ng/mL activin A group; (**C**) Caspase-3 activity assay was used to test the relative caspase-3 activity of the control. The data are presented as the mean ± standard deviation. *** *p* < 0.001 versus the 100 ng/mL activin A group.

Flow cytometry and caspase-3 activity assays showed that the apoptotic rate did not obviously increase when the activin A concentration was between 0 and 50 ng/mL, thereby stimulating H9c2 cells for 24 h. However, the apoptotic rate dramatically increased (*p* < 0.001) when the activin A concentration increased to 100 ng/mL (Figure 5).

### 2.7. Use of High Concentration (100 ng/mL) of Activin A to Show that the Activin A–Follistatin System Regulated ERS-Mediated Cardiomyocyte Apoptosis In Vitro

**Figure 6:**
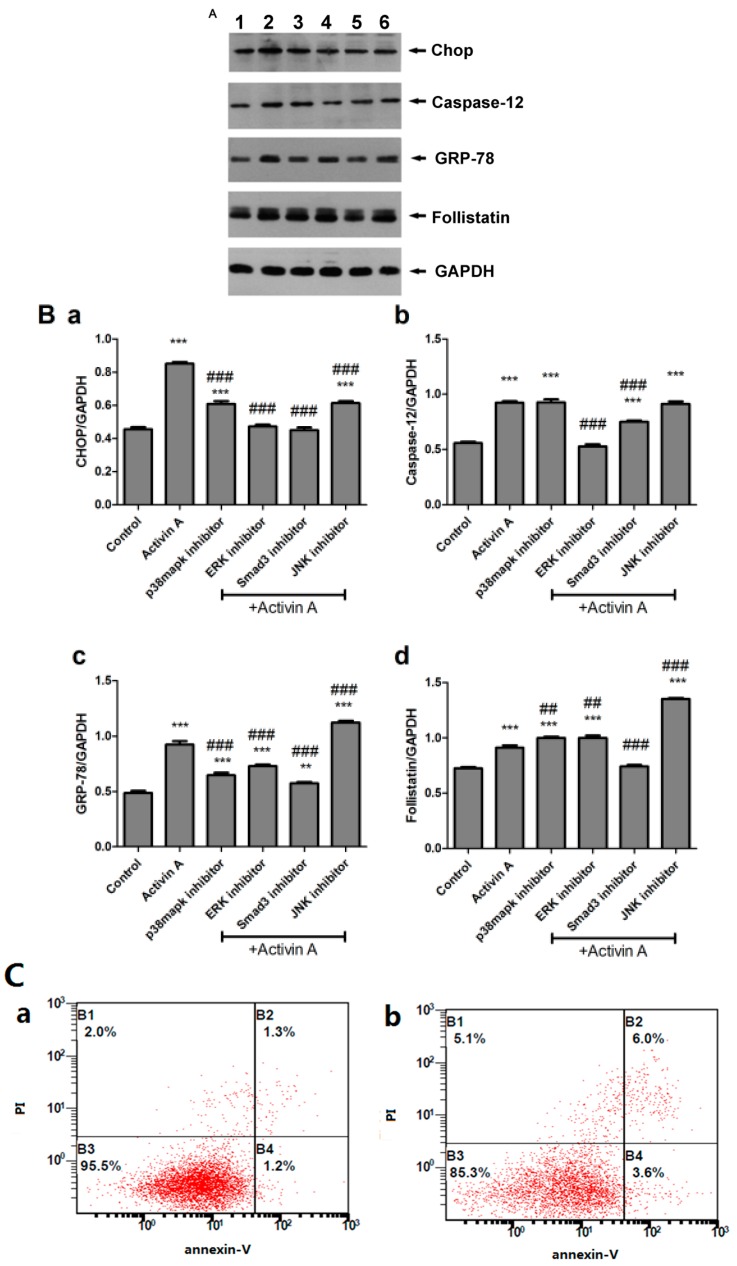

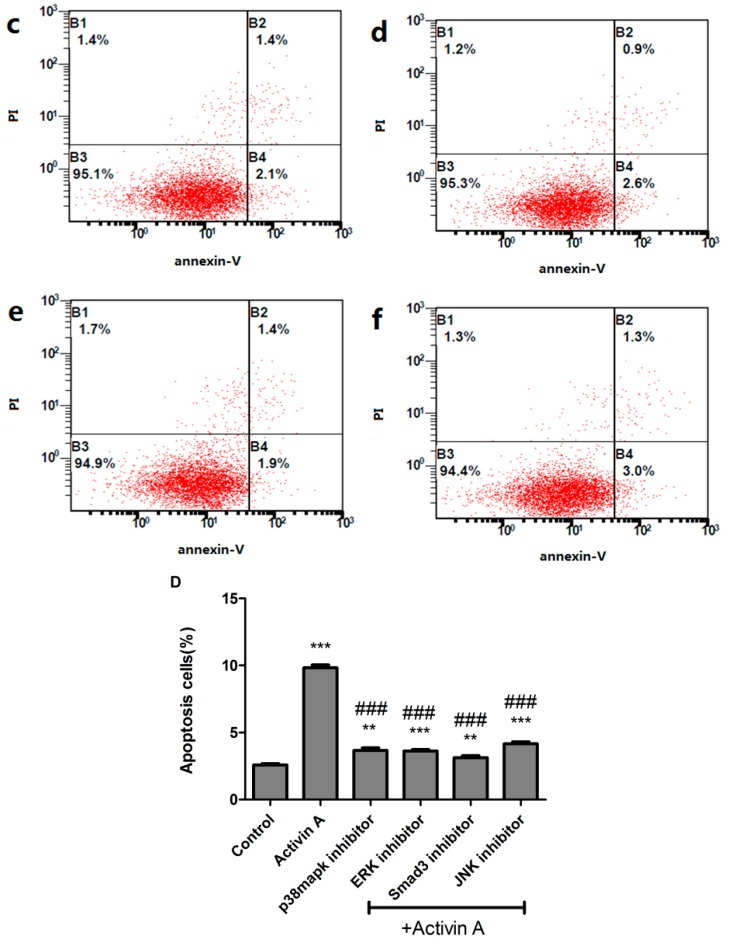
Expression levels of follistatin and endoplasmic reticulum stress (ERS)-related molecules in activin A-stimulated H9c2 cells. (**A**) Western blot was performed to test the expression of follistatin and ERS-related molecules. Proteins were extracted from H9c2 cells, separated by sodium dodecyl sulfate–polyacrylamide gel electrophoresis, and immunoblotted sequentially with antibodies. (1) Control, (2) activin A (100 ng/mL), (3) activin A (100 ng/mL) + p38MAPK inhibitor, (4) activin A (100 ng/mL) + ERK inhibitor, (5) activin A (100 ng/mL) + Smad3 inhibitor, (6) activin A (100 ng/mL) + JNK inhibitor; (**B**) The graph showed the result of densitometric quantification of proteins relative to GAPDH as an internal control. (**a**) CHOP, (**b**) caspase-12, (**c**) GRP-78, (**d**) Follistatin; (**C**) H9c2 cells were stained with PI and Annexin V-FITC and sorted by fluorescence-activated flow cytometry. (**a**) Control, (**b**) activin A (100 ng/mL), (**c**) activin A (100 ng/mL) + p38MAPK inhibitor, (**d**) activin A (100 ng/mL) + ERK inhibitor, (**e**) activin A (100 ng/mL) + Smad3 inhibitor, (**f**) activin A (100 ng/mL) + JNK inhibitor; (**D**) Graph presented the apoptotic rate of H9c2 cells. The data are presented as the mean ± standard deviation. ** *p* < 0.01, *** *p* < 0.001 versus the control group; ^##^ *p* < 0.01, ^###^ *p* < 0.001 versus the activin A group.

H9c2 cells were cultured with activin A and inhibitors for 24 h. Western blot results showed that the expression levels of CHOP, caspase-12, GRP-78, and follistatin increased obviously in the activin A group. The expression levels of CHOP and GRP-78 decreased significantly in the p38 mitogen-activated protein kinase (p38MAPK) inhibitor + activin A group compared with the activin A group (*p* < 0.001). The expression levels of CHOP, caspase-12, and GRP-78 decreased obviously in the extracellular regulated protein kinases (ERK) inhibitor + activin A group compared with the activin A group (*p* < 0.001). Only the expression of CHOP reduced remarkably in the JNK inhibitor + activin A group compared with the activin A group (*p* < 0.001). The follistatin expression appeared to increase in the three signaling pathway-inhibiting groups compared with the activin A group. The expression of ERS-related molecules and follistatin decreased significantly in the Smad3 inhibitor + activin A group compared with the activin A group (Figure 6A,B). The apoptotic rate was measured by flow cytometry. It dramatically increased in the 100 ng/mL activin A group compared with the control group (*p* < 0.001). The apoptotic rates obviously reduced in mitogen-activated protein kinase (MAPK) inhibitors + activin A groups and Smad3 inhibitor + activin A group compared with the activin A group (*p* < 0.001).

### 2.8. Concentration and Acting Time of Ang II-Stimulated H9c2 Cell Apoptosis

**Figure 7:**
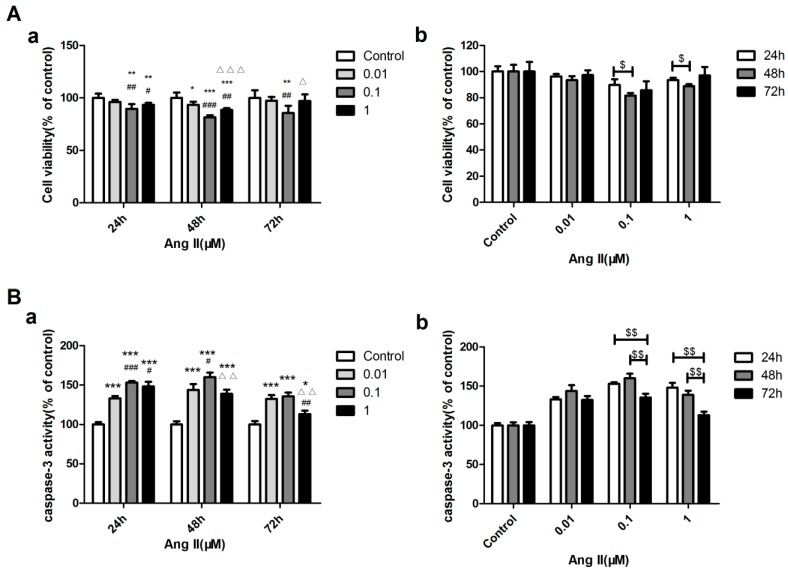
Concentration and acting time of Ang II-stimulated H9c2 cell apoptosis. (**A**) Cell viability was assessed using the MTT assay. H9c2 cells were seeded at a density of 5 × 10^3^ cells per well into 96-well plates. (**a**) Effect of Ang II on the viability of H9c2 cells cultured with different concentrations (0.01, 0.1, 1 μM·mol/L) for the same time; (**b**) Effect of Ang II on the viability of H9c2 cells cultured with the same concentration for different times (24, 48 or 72 h); (**B**) Caspase-3 activity assay was used to test the relative caspase-3 activity of the control. (**a**) Effect of Ang II on the caspase-3 activity of H9c2 cells cultured with different concentrations (0.01, 0.1, 1 μM·mol/L) for the same time; (**b**) Effect of Ang II on the caspase-3 activity of H9c2 cells cultured with the same concentration for different times (24, 48 or 72 h). The data are presented as the mean ± standard deviation. * *p* < 0.05, ** *p* < 0.01, *** *p* < 0.001 versus the control group; ^#^ *p* < 0.05, ^##^ *p* < 0.01, ^###^ *p* < 0.001 versus the 0.01 μM Ang II group; ^Δ^ *p* < 0.05, ^ΔΔ^ *p* < 0.01, ^ΔΔΔ^ *p* < 0.001 versus the 0.1 μM Ang II group, ^$^ *p* < 0.05, ^$$^ *p* < 0.01.

Ang II was used to induce cardiomyocyte apoptosis. The MTT method was used to test the cell viability and caspase-3 activity assay was performed to detect apoptosis after stimulating H9c2 cells with 0.01, 0.1, and 1 μM concentrations of Ang II for 24, 48, and 72 h, respectively. Subsequently, 0.1 μM Ang II began to reduce the cell viability obviously after 24 h (*p* < 0.01). The cell viability was found to decrease dramatically with 0.1 μM Ang II stimulating H9c2 for 48 h (Figure 7A). However, the caspase-3 activity assay showed that stimulating H9c2 with 0.1 μM Ang II for 24 and 48 h had the same effect (Figure 7B). Therefore, 0.1 μM Ang II was used to establish the cell apoptosis model. In the foregoing experiments, effects of activin A and AngII were seen within 24 h. Therefore, in the following experiments, costimlulation with activin A and AngII was evaluated after 24 h.

### 2.9. Effect of Low Concentration of Activin A on Ang II-Induced Cardiomyocyte Apoptosis and Its Relationship with ERS

**Figure 8:**
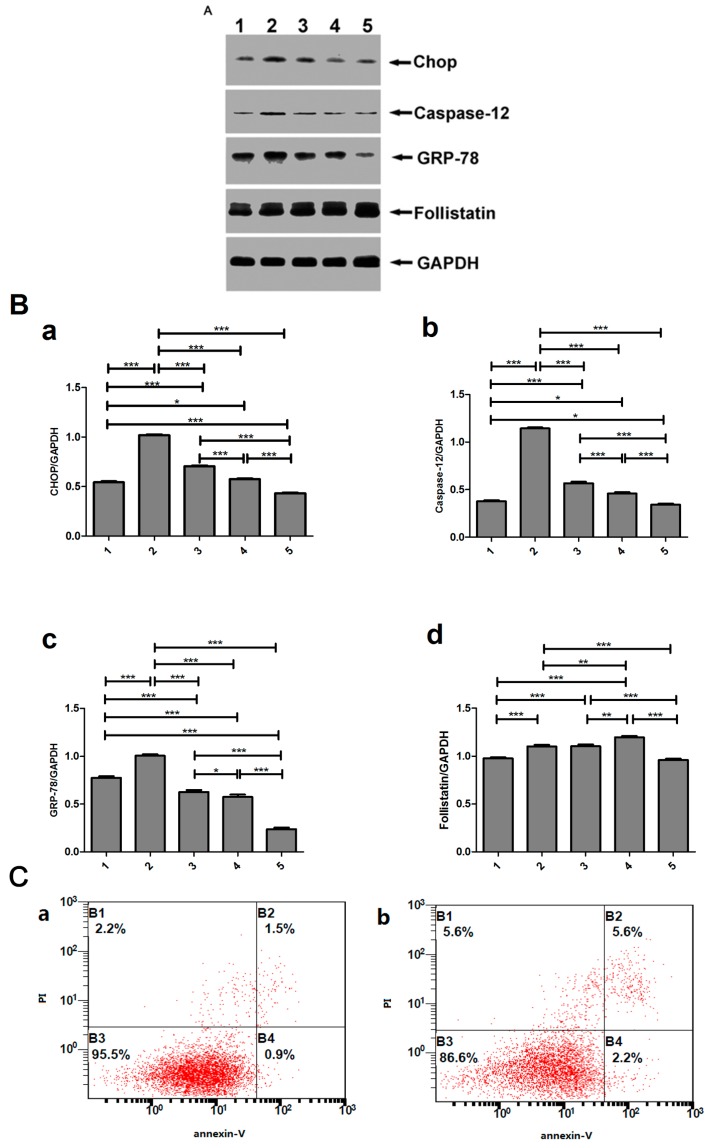

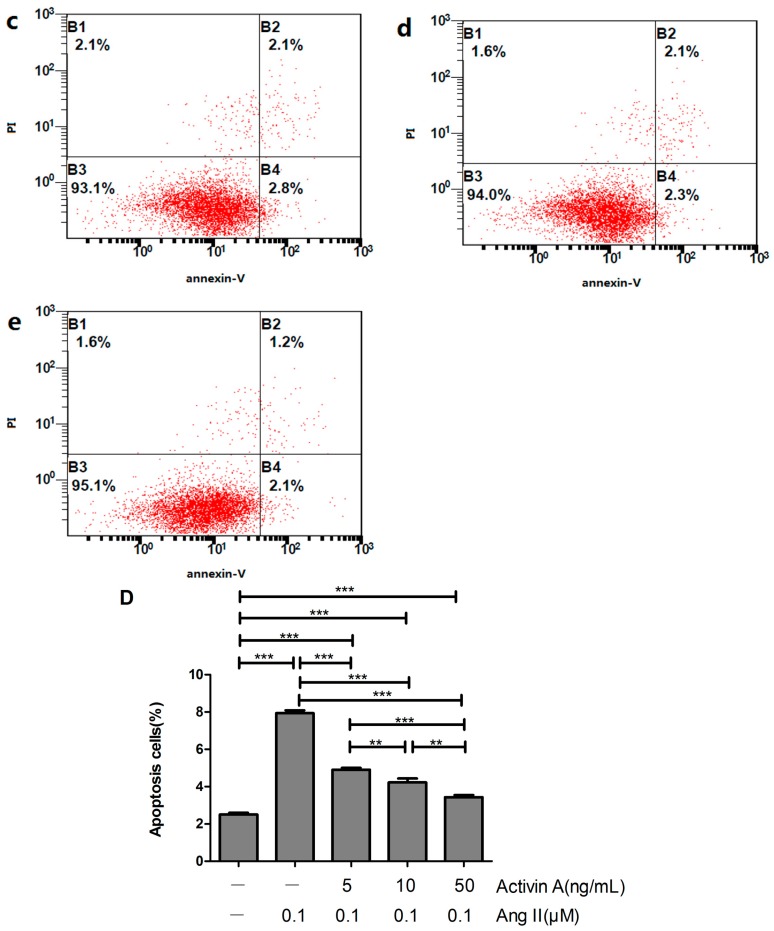
Expression levels of follistatin and ERS-related molecules in Ang II-stimulated H9c2 cells cultured with a low concentration of activin A. (**A**) Western blot was performed to determine the expression of follistatin and ERS-related molecules. Proteins were extracted from H9c2 cells, separated by sodium dodecyl sulfate−polyacrylamide gel electrophoresis, and immunoblotted sequentially with antibodies. (1) Control, (2) Ang II (0.1 μM), (3) activin A (5 ng/mL) + Ang II (0.1 μM), (4) activin A (10 ng/mL) + Ang II (0.1 μM), and (5) activin A (50 ng/mL) + Ang II (0.1 μM); (**B**) The graph showed the result of densitometric quantification of proteins relative to GAPDH as an internal control. (**a**) CHOP, (**b**) caspase-12, (**c**) GRP-78, (**d**) Follistatin; (**C**) H9c2 cells were stained with PI and Annexin V–FITC and sorted by fluorescence-activated flow cytometry. (**a**) Control, (**b**) Ang II (0.1 μM), (**c**) activin A (5 ng/mL) + Ang II (0.1 μM), (**d**) activin A (10 ng/mL) + Ang II (0.1 μM), (**e**) activin A (50 ng/mL) + Ang II (0.1 μM); (**D**) Graph presented the apoptotic rate of H9c2 cells. The data are presented as the mean ± standard deviation. * *p* < 0.05, ** *p* < 0.01, *** *p* < 0.001.

H9c2 cells were cultured with 0.1 μM Ang II and activin A for 24 h. The Western blot result showed that the expression levels of ERS-related molecules obviously increased in the 0.1 μM Ang II group compared with the control group. As activin A concentrations elevated to 50 ng/mL, the expression levels of ERS-related molecules gradually reduced compared with the 0.1 μM Ang II group (*p* < 0.001). However, the expression of follistatin increased when the concentration of activin A was below 50 ng/mL, and apparently decreased when the concentration was up to 50 ng/mL, compared with the 0.1 μM Ang II group (*p* < 0.001) (Figure 8A,B). Apoptotic rate was measured by flow cytometry, and the result showed that apoptosis gradually reduced as activin A concentrations elevated to 50 ng/mL (Figure 8C,D).

## 3. Discussion

Recently, the focus of studies investigating the HF mechanism has moved from neuroendocrine activation to cytokines, including tumor necrosis factor-α, interleukin-1 (IL-1), IL-6, endothelin family, TGF-β, and so forth, which are involved in ventricular remodeling [31,32,33,34]. Activin A is a secretory multifunctional cell growth factor belonging to the TGF-β superfamily. Follistatin directly binds to activin A extracellularly [16], inhibiting the physiological action of activin A. Therefore, this study aimed to explore the role of activin A and follistatin as a whole in the occurrence and development of HF.

Previous studies suggested that activin A was associated with the occurrence and severity of HF [35,36], but the underlying mechanism was unclear. Previous studies on activin A mainly focused on promoting myocardial hypertrophy and fibrosis [37,38,39]. Recently, an extremely important study illustrated the role of activin A–follistatin system in myocardial cell apoptosis [22]. The expression levels of activin A and follistatin like-3 (Fstl-3) had an increasing tendency, while the expression of follistatin mRNA had no obvious change in the acute cardiac injury model. Activin A inhibited cardiomyocyte apoptosis induced by hypoxia-reoxygenation injury in vitro, and Fstl-3 had an antagonistic effect on it. However, the aforementioned study could only suggest that activin A inhibited myocardial cell apoptosis in acute injuries and prevented a heart attack. Whether activin A has the same protective role in myocardial cell apoptosis after chronic damage such as HF after MI needs to be confirmed.

A rat model of HF was established after MI in this study. It also showed [36] that the serum activin A gradually increased during MI and was positively correlated with the serum BNP. The Western blot results revealed that the expression levels of activin A and BNP of noninfarction areas of the left ventricle increased in the MI groups. The longer the MI, the more obvious the expression of two proteins. The aforementioned data suggested that activin A could serve as a new cardiac biomarker for diagnosing and evaluating HF. An interesting finding was that the level of serum follistatin was significantly elevated in rats with four-week and eight-week MI (Figure 2A). The ratios of the activin A–follistatin system in the serum of all the groups were nearly unchanged (Figure 2B). The Western blot results showed that, in the left ventricular noninfarction area, the expression of activin A increased as MI was prolonged and the expression of follistatin had no obvious change until four weeks after MI (*p* < 0.001), and the expression in the four-week MI group was almost the same as in the eight-week MI group (Figure 4). We also calculated the ratio of activin A–follistatin system in the myocardial tissue. It showed that the ratios of all MI groups increased compared with the SO groups, and the increases were more apparent as MI was prolonged (Figure 4C). The aforementioned data suggested that follistatin might not be invariably expressed in the development of HF. However, it would obviously be expressed in the mid/late-stage of HF. Activin A began to be highly expressed in the early stage of HF. This might be related to the self-feedback regulatory mechanism of the body to maintain a balance of the activin A–follistatin system. When activin A was over-expressed, the activin A–follistatin system balance would break, and the body would later produce follistatin to neutralize activin A to delay the progress of HF. At last, the system restored the balance.

The endoplasmic reticulum pathway has been discovered as a new apoptotic pathway in recent years. ERS occurs under adverse conditions [40]. It was known that moderate ERS, as the cell’s initial response to stress, reduced apoptosis; conversely, persistent or severe ERS promoted apoptosis [23]. Hence, a close relationship exists between ERS and myocardial cell apoptosis. Studies have shown that various adverse factors, such as hypoxia and ischemia, cause excessive ERS, inducing myocardial cell apoptosis through CHOP, caspase-12, and JNK apoptosis pathways [24,41,42]. Assuming in our research that over-expression of activin A influences myocardial cell apoptosis via ERS, animal models were established to observe whether the imbalance of the activin A–follistatin system existed and participated in ERS-mediated myocardial cell apoptosis during different periods of HF. Then, cell models were constructed to investigate the influence of activin A with different concentrations on cardiomyocyte apoptosis and its relationship with ERS by measuring caspase-3 activity and apoptotic rate through flow cytometry and the expressions of CHOP, caspase-12 and GRP-78 represented ERS-mediated apoptosis through western blot.

Furthermore, the immunohistochemical staining of rats showed that the expression of caspase-3 and CHOP in the infarction and noninfarction areas of the left ventricle significantly increased in the eight-week MI group, which confirmed that cell apoptosis existed in the myocardial tissue, the ERS pathway played a role in HF, and the activin A–follistatin system probably participated in the myocardial cell apoptosis through the ERS pathway (Figure 3). GRP-78 is a member of the heat-shock protein 70 (HSP70) family. It is responsible for cellular homeostasis. It binds with immunoglobulin heavy chain-binding protein to form ER molecular chaperones, which participate in unfolded protein response (UPR), the most important signaling mechanism inducing ERS [43]. The Western blot results showed that GRP-78 and CHOP were highly expressed one week after MI, whereas caspase-12 was significantly expressed four weeks after MI. The expression levels of activin A were higher in one-week, four-week, and eight-week MI groups than in the control group and increased as MI prolonged (Figure 4). These findings suggested that over-expressed activin A probably induced excessive UPR and ERS, leading to myocardial cell apoptosis and accelerating the process of HF. Although the expression of follistatin increased four weeks after MI, it was nearly unchanged in the four- and eight-week MI groups (Figure 4). It indicated that follistatin increased to maintain the balance of activin A–follistatin system after MI to attenuate the myocardial cell apoptosis induced by activin A and delay the process of HF.

The results above somewhat contradict the previous research [21]. Activin A was shown to play a suppressive role in myocardial cell apoptosis in previous studies but a stimulatory role in our study. Therefore, it was hypothesized that the effect of activin A on myocardial cell apoptosis was related to its time and level of expression.

Cell experiments were designed to prove the present hypothesis. Different concentrations of activin A were used to stimulate H9c2 cells (Figure 5), and a high concentration of activin A (100 ng/mL) was found to induce cardiomyocyte apoptosis. H9c2 cells were incubated in a culture medium with the inhibitors of p38MAPK, ERK, and JNK signaling pathways, and Smad3 inhibitor was added separately. Then, 100 ng/mL activin A was used to induce cardiomyocyte apoptosis. Finally, the expression levels of ERS pathway-related molecules and follistatin were tested. The results suggested that a high concentration of activin A could activate p38MAPK, ERK, and JNK signaling pathways and induce cardiomyocyte apoptosis through the ERS pathway. The expression levels of follistatin in the activin A group, p38MAPK inhibitor + activin A group, ERK inhibitor + activin A group, and JNK inhibitor + activin A group increased more significantly than those in the control group, but obviously decreased in the Smad3 inhibitor + activin A group, probably suggesting that the expression of follistatin was directly dependent on the expression of activin A through the Smads signaling pathway (Figure 6).

The cardiomyocyte apoptosis model was induced by Ang II to further investigate the effect of activin A with a low concentration and its relationship with ERS (Figure 8). The concentration and acting time of Ang II were detected by the MTT method and caspase-3 activity assay (Figure 7). The reason why Ang II was used to induce cardiomyocyte apoptosis was that when HF happened, the renin–angiotensin–aldosterone system (RAAS) system was activated, increasing Ang II dramatically, as is shown in Figure 2. Using Ang II to induce apoptosis could be better to imitate the environment in vivo in HF after MI. The results indicated that low concentrations of activin A could inhibit Ang II-induced cardiomyocyte apoptosis through the ERS pathway. The higher the concentrations of activin A, the weaker the ERS and the less the cardiomyocyte apoptosis (Figure 8). The aforementioned data indicate that the effects of different concentrations of activin A on cardiomyocyte were different. Activin A could inhibit cardiomyocyte apoptosis at a low concentration, but induce cardiomyocyte apoptosis at a high concentration, probably via the ERS pathway (Figure 6 and Figure 8). The expression of follistatin began to increase when the concentration of activin A was up to 10 ng/mL but statistically decreased when the concentration of activin A was up to 50 ng/mL. Therefore, the expression level of follistatin was likely relative to the concentration of activin A, and the specific quantitative relation or specific concentrations of activin A and follistatin need to be further investigated.

## 4. Materials and Methods

All experimental procedures were approved by the Ethical Board Review of China-Japan Union Hospital of Jilin University (permission code: 2016ks017, permission date: 3 January 2016).

### 4.1. Materials

The female Wistar rats with the body weight of 200–220 g were obtained from the Center for Laboratory Animals, Medical College, Jilin University, Changchun, China. The H9c2 cells, a rat cardiomyoblast line, were purchased from American Type Culture Collection (Manassas, VA, USA).

All components of the cell culture medium were purchased from Gibco (Invitrogen Life Technologies, Carlsbad, CA, USA). Enzyme-linked immunosorbent assay (ELISA) kits, human/mouse/rat activin A βA subunit biotinylated monoclonal antibody, and recombinant human, mouse, and rat activin A were purchased from R&D Systems (Abingdon, UK). The rabbit anti-rat follistatin polyclonal antibody and rabbit anti-rat brain natriuretic peptide (BNP) polyclonal antibody were obtained from Abcam plc (Cambridge, UK). The anti-caspase-12 (1611), which is a rat monoclonal antibody; anti-GRP 78 (76-E6), which is a rat monoclonal antibody; anti-GADD 153 (B-3) (CHOP), which is a mouse monoclonal antibody; and rabbit anti-rat caspase-3 polyclonal antibody were purchased from Santa Cruz Biotechnology, Inc. (Dallas, TX, USA).

MTT (3-(4,5-dimethylthiazol-2-yl)-2,5-diphenyl-tetrazoliumbromide) and glyceraldehyde-3-phosphate dehydrogenase (GAPDH) were obtained from Sigma-Aldrich (St. Louis, MO, USA). The Annexin V-fluorescein isothiocyanate (FITC) apoptosis detection kit was purchased from BioVision, Inc. (Milpitas, CA, USA). Angiotensin II (Ang II) was obtained from Phenix Research Products (Candler, NC, USA).

SB203580 (p38 mitogen-activated protein kinase (p38MAPK) inhibitor), PD98059 (extracellular regulated kinase (ERK) inhibitor), SP600125 (JNK inhibitor), and SIS3 (Smad3 inhibitor) were purchased from Merck Millipore (Darmstadt, Germany).

The caspase-3 activity colorimetric assay kit was purchased from BestBio (Beijing, China).

### 4.2. Establishment of the Rat Model with Heart Failure (HF)

The rats were randomly assorted into four groups: sham-operated, one-week MI, four-week MI, and eight-week MI with six rats in each group. The diethyl ether-anesthetized rats were fixed on an operating table. The heart was exposed by opening the thoracic cavity, and the left anterior descending coronary artery was ligated. The rats of the SO group underwent the same procedure except for the suture under the coronary artery that was left untied. The heart was then placed in its original position, and the thorax was immediately closed. All rats had full access to a standard diet and tap water under a room temperature of (21 ± 1) °C with a 12 h light/dark cycle in different weeks.

### 4.3. Hemodynamic Investigation

Different groups of postoperative rats were fed for one week, four weeks, and eight weeks, respectively; weighed; and anesthetized by intraperitoneal injection of 3% pentobarbital sodium (30 mg/kg). Then, the right common carotid arteries of the rats were separated, and a tube, connected to the pressure transducers and the AP-621G carrier amplifier, was inserted into the left ventricle of each rat through the right common carotid artery. Hemodynamic characteristics were recorded through the BCL-400 biological function experimental system.

### 4.4. ELISA

The ELISA assay was performed strictly according to the kit instructions. The optical density (OD) values were measured at 490 nm using a spectrophotometric microplate reader. The calibration curves were drawn with OD values as the vertical axis (*Y*) and activin A, follistatin, or BNP standard concentrations as the abscissa axis (*X*). The concentration data of activin A, follistatin, or BNP samples could be obtained by OD value conversion on the basis of calibration curves.

### 4.5. Immunohistochemical Staining

Immunohistochemical staining for activin A, follistatin, CHOP, and caspase-3 was conducted on the myocardial tissue. The myocardial tissue of the left ventricle was deparaffinized, rehydrated in a graded series of alcohol solutions, and washed twice with distilled water. The sections were incubated with endogenous peroxidase blocked in 50 μL of 3% H_2_O_2_ at room temperature for 10 min, and then washed with phosphate-buffered saline (PBS) (with the pH of 7.4) three times for 3 min each. The 2% bovine serum albumin in PBS was added and incubated at room temperature for 30 min, and the sections were washed once in PBS. Activin A, follistatin, CHOP, and caspase-3 antibodies were added and incubated at 4 °C overnight. The activin A, follistatin, CHOP, and caspase-3 proteins were assayed using an ultrasensitive SP kit. The sections were counterstained with hematoxylin. The incubation of tissue sections with immunoglobulin G (IgG) from normal rabbit served as a negative control. The method was previously described by Wen-qi et al. [44]. The border zones were used to take photos. The infarction and noninfarction areas and border zones could be clearly seen compared to the SO groups in the immunohistochemical sections, so the border zones can be directly examined under low power lens, and then change into high power lens.

### 4.6. Cell Culture

H9c2 cells were cultured in Dulbecco’s modified Eagle’s medium (1×) with high glucose (4500 mg/L), l-glutamine (4.0 mM), and sodium pyruvate. Then, 10% fetal bovine serum, 100 U/mL penicillin, and 100 mg/mL streptomycin were added to the culture medium in a moist atmosphere with 5% CO_2_ at 37 °C. The H9c2 cells were inoculated in six-well plates, cell culture flasks of 25 cm^2^, and culture dishes of 100 × 20 mm^2^ in the course of logarithmic growth. When the cells in the plates, flasks, or dishes reached 80% confluence, other experiments were performed in vitro.

### 4.7. MTT Assay

This assay was based on the transformation of tetrazolium salt into an insoluble formazan salt (MTT) by active mitochondria. The H9c2 cells were seeded at the density of 5 × 10^3^ cells per well into 96-well plates calculated by the direct smear counting method. After 24 h, they were divided into different groups with different Ang II concentrations (0.01–1 μM) for another 24, 48, or 72 h. Then, MTT (5 mg/mL) was added to the cells (20 μL/well) for 4 h. Finally, the medium was removed from the wells, and dimethyl sulfoxide was added into the wells to resolve the formazan salt (150 μL/well). The plates were then shaken at room temperature for 10–30 min. The OD value was measured at 490 nm using a spectrophotometer. The viable count percentage was defined as the relative OD value of the treated cells versus the untreated control cells. The method was previously described by Chunyan Yang et al. [45].

### 4.8. Flow Cytometry

The Annexin V apoptosis detection kit could differentiate apoptosis from necrosis through Annexin V–FITC and propidium iodide (PI) staining. Apoptosis was analyzed by flow cytometry. H9c2 cells (1 × 10^5^–5 × 10^5^) were induced with different concentrations (6.25, 12.5, 25, 50, and 100 ng/mL) of activin A and 0.1 μM Ang II for 24 h. They were collected using ethylenediaminetetraacetic acid-free trypsin on the ice and washed once with serum-containing media. Then, they were washed with ice-cold PBS and centrifuged at 1000× *g* for 5 min. They were resuspended in 500 μL of 1× binding buffer, mixed with 5 μL of Annexin V–FITC and 5 μL of PI (50 μg/mL, optional), and incubated at room temperature for 5 min in the dark. Annexin V–FITC binding was analyzed by flow cytometry (E_x_ = 488 nm; E_m_ = 530 nm) using an FITC signal detector (usually FL1) and PI staining using a phycoerythrin emission signal detector (usually FL2). The cells stained only with Annexin V-FITC were regarded as early apoptosis, and the cells stained with both PI and Annexin V-FITC were regarded as late apoptosis.

### 4.9. Assay of Caspase-3 Activity

A caspase-3 activity colorimetric assay kit was used to confirm the existence of apoptosis. The assay was carried out strictly according to the kit instructions after preparing cell lysates from apoptotic cells.

### 4.10. Western Blot

After preparing and collecting the protein samples, the concentration of each protein sample was measured by the Bradford colorimetric method to ensure that each protein sample was consistent.

The protein (30 mg) was subjected to sodium dodecyl sulfate (SDS)-polyacrylamide gel electrophoresis after boiling the samples with the SDS sample buffer (2% SDS, 10% glycerol, 5% 2-mercaptoethanol, 0.002% bromophenol blue, and 62.5 mM Tris-HCl, pH 6.8). The proteins were then transferred onto a cellulose nitrate membrane, blocked, and probed with primary antibodies against follistatin, CHOP, caspase-12, GRP-78, and GAPDH. Next, the membrane was incubated with horseradish peroxidase-conjugated goat anti-rabbit IgG. Finally, the bound antibody complexes were measured using a chemiluminescence reagent (ECL Plus; Amersham Pharmacia Biotech, Tokyo, Japan).

### 4.11. Statistical Analyses

All the trials were performed at least three times. The data were recorded as the mean ± standard deviation. Different treatment groups were compared using analysis of variance, followed by the Dunnett post hoc test. The percent changes were analyzed using the Kruskal–Wallis H test. A *p*-value < 0.05 was considered statistically significant.

## 5. Conclusions

This study illustrated that the activin A–follistatin system was involved in endoplasmic reticulum stress (ERS)-mediated myocardial cell apoptosis in heart failure (HF). Activin A can become a new cardiac biomarker for diagnosing HF in the future. More studies should be conducted to explore the methods that inhibit the expression of activin A or promote the expression of follistatin, ultimately downregulating the ratio of activin A to follistatin in the body, which may be a new target to improve ventricular remodeling and HF.

## Figures and Tables

**Table 1:** The sizes of the hearts of rats. The long diameter and short diameter of hearts were measured using a vernier caliper. ** *p* < 0.01, compared with the sham-operated (SO) group. ^#^ *p* < 0.05, ^##^ *p* < 0.01, compared with the one-week myocardial infarction (MI) group. ^Δ^ *p* < 0.05 compared with the four-week MI group.

Size of Heart (cm)	SO			MI		
	1 W	4 W	8 W	1 W	4 W	8 W
long diameter	1.383 ± 0.183	1.483 ± 0.075	1.533 ± 0.081	1.4 ± 0.19	1.65 ± 0.055 **^,##^	1.75 ± 0.084 **^,##,Δ^
short diameter	0.15 ± 0.105	1.233 ± 0.052	1.217 ± 0.117	1.067 ± 0.121	1.3 ± 0.167 ^#^	1.367 ± 0.121 ^##^

**Table 2:** Morphological and hemodynamic characteristics of rats. BW: body weight. HW: heart weight. LVHW: left ventricular heart weight. HR: heart rate. SBP: systolic blood pressure. DBP: diastolic blood pressure. +dp/dt: maximal rate of rise of blood pressure in ventricle chamber. −dp/dt: maximal rate of rise of blood pressure in ventricle chamber. LVESP: left ventricular end systolic pressure. LVEDP: left ventricular end diastolic pressure. * *p* < 0.05, ** *p* < 0.01, *** *p* < 0.001, compared with the SO group. ^#^ *p* < 0.05, ^##^ *p* < 0.01, ^###^ *p* < 0.001, compared with the one-week MI group. ^Δ^ *p* < 0.05, ^ΔΔ^ *p* < 0.01, compared with the four-week MI group.

Characteristics	SO			MI		
	1 W	4 W	8 W	1 W	4 W	8 W
HW/BW (mg/g)	3.20 ± 0.098	3.21 ± 0.130	3.19 ± 0.163	3.86 ± 0.217 ***	3.80 ± 0.219 ***	3.52 ± 0.158 **^,#,Δ^
LVW/BW (mg/g)	2.24 ± 0.130	2.22 ± 0.145	2.18 ± 0.192	2.68 ± 0.105 ***	2.50 ± 0.206 *	2.46 ± 0.155 *^,#^
HR (/min)	435 ± 31.45	437.33 ± 31.10	430 ± 52.31	413.83 ± 50.70	410.66 ± 68.24	353.67 ± 37.47 *^,#^
SBP (mmHg)	98.97 ± 12.67	98.89 ± 15.13	97.03 ± 13.06	89.79 ± 14.01	81.84 ± 19.14	63.78 ± 5.61 ***^,##^
DBP (mmHg)	59.96 ± 3.67	76.34 ± 17.73	66.79 ± 10.87	75.22 ± 15.72 *	69.97 ± 20.59	53.06 ± 5.19 *^,##^
LVSP (mmHg)	115.98 ± 3.53	110.48 ± 18.51	105.15 ± 12.19	63.12 ± 8.96 ***	60.06 ± 3.96 ***	47.90 ± 7.50 ***^,##,ΔΔ^
LVEDP (mmHg)	3.82 ± 9.28	3.745 ± 10.04	3.665 ± 9.84	22.76 ± 7.19 **	23.97 ± 8.35 **	24.15 ± 4.83 **
+dp/dtmax (mmHg/s)	5455 ± 443.92	5373.33 ± 842.29	5465 ± 974.77	2720 ± 248.83 ***	2498.33 ± 251.42 ***	2070 ± 140.28 ***^,###,ΔΔ^
−dp/dtmax (mmHg/s)	3885 ± 321.66	4460 ± 989.86	4146.667 ± 817.32	2171.667 ± 216.92 ***	2171.667 ± 172.55 ***	1850 ± 235.96 ***^,#,Δ^
